# Supplementary material for: Human Gut Faecalibacterium prausnitzii Deploys a Highly Efficient Conserved System To Cross-Feed on β-Mannan-Derived Oligosaccharides
Source: mBio. 2021 Jun 1;12(3):e03628-20. doi: 10.1128/mBio.03628-20 (PMC8262883; doi:10.1128/mBio.03628-20)

**Figure S1**. ITC analysis of *Fp*MOBP binding to mannohexaose and cellohexaose. **a,** Binding to mannohexaose using a MicroCal ITC_200_ microcalorimeter. The top panel shows the thermogram, whereas the bottom graph depicts the binding isotherms and one set of equivalent binding sites model fits to the data (solid lines).The analyses were performed using 2.5 mM of ligand in the syringe and 22.5 µM *Fp*MOBP in the cell. **b,** Binding of *Fp*MOBP to mannohexaose and cellohexaose using a MicroCal VP-ITC system. The panel shows the thermograms of 22.5 µM µM *Fp*MOBP titrated with either 2.5 mM mannohexaose (black line) or 2.5 mM cellohexaose (blue line). When comparing the two thermograms it is apparent that cellohexaose binding to *Fp*MOBP is not detected.


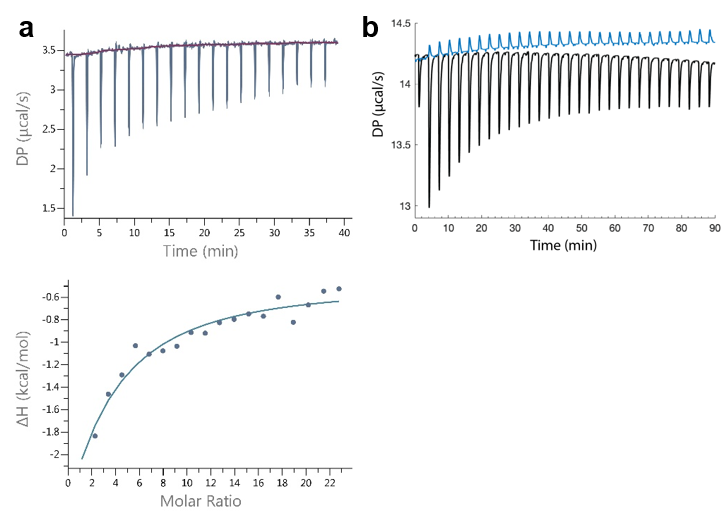

Supplement: FIG S1 [file mbio.03628-20-sf001.docx]
