# Supplementary material for: Human Gut Faecalibacterium prausnitzii Deploys a Highly Efficient Conserved System To Cross-Feed on β-Mannan-Derived Oligosaccharides
Source: mBio. 2021 Jun 1;12(3):e03628-20. doi: 10.1128/mBio.03628-20 (PMC8262883; doi:10.1128/mBio.03628-20)

**Figure S2.** Hydrolysis of galactosylated β-MOS and galactomannan by *Fp*GH113 and time course activity. **a,** HPAEC-PAD analysis of the products generated by *Fp*GH113 and *Fp*GH36 incubated with Gal_2_M_5_ for 16h using 10 mM sodium phosphate, pH 5.8. A reaction with the reducing end mannose-releasing exo-oligomannosidase *Ri*GH113 was used as a control. Assignment of peaks not corresponding to the standards is based on the MALDI-ToF MS analysis shown in **b**. In the cartoon representation of Gal_2_M_5_, the orange and green dashed lines indicate the linkage cleaved by *Fp*GH36 and *Fp*GH113/*Ri*GH113, respectively, as deduced by the MALDI-ToF spectra. **c,** A time course of *Fp*GH113 acting on mannopentaose. **d,** HPAEC analysis of the activity of *Fp*GH113 on intact carob galactomannan (CGM). The data shown are representative of independent triplicates. Abbreviations: M_1_, mannose, M_2_, mannobiose; M_3_, mannotriose; M_4_, mannotetraose; M_5_, mannopentaose; M_6_, mannohexaose, Gal_1_, galactose; Gal_2_M_4_, digalactosylmannotetraose; Gal_2_M_5_, digalactosylmannopentaose; Hex, hexose.

[figure on next page]


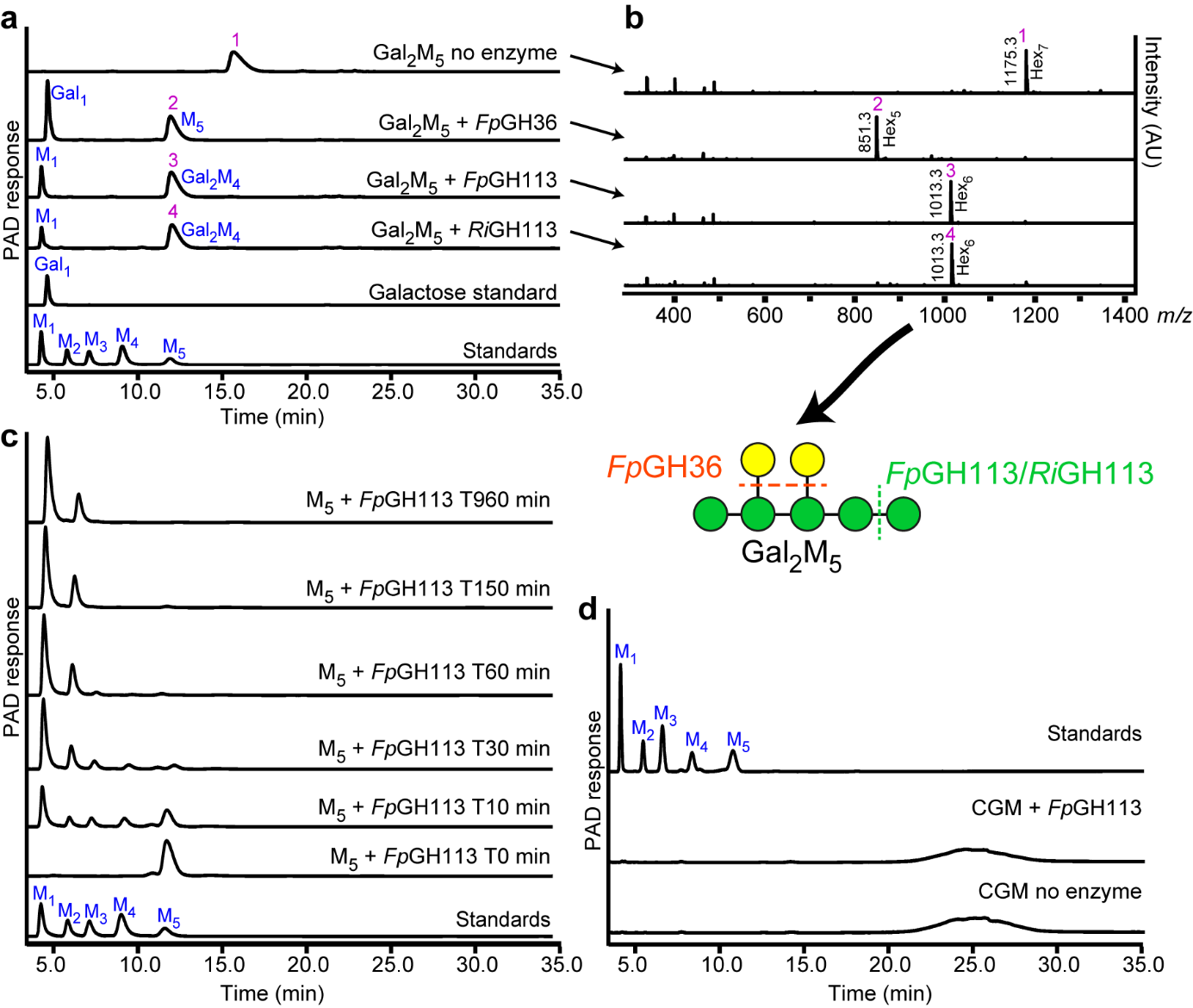

Supplement: FIG S2 [file mbio.03628-20-sf002.docx]
