# Supplementary material for: Human Gut Faecalibacterium prausnitzii Deploys a Highly Efficient Conserved System To Cross-Feed on β-Mannan-Derived Oligosaccharides
Source: mBio. 2021 Jun 1;12(3):e03628-20. doi: 10.1128/mBio.03628-20 (PMC8262883; doi:10.1128/mBio.03628-20)

**Figure S3**. SCFAs detected in single cultures (blue bars) of *F. prausnitzii* or in co-cultivation with either heat-treated (orange bars) or untreated (green bars) *B. ovatus* cells. The experiment was conducted in M2 medium supplemented with KGM at 37 °C. *B. ovatus* V975 was pre-grown in M2GSC and incubated until OD of about 0.5 was reached. Cultures in Hungate tubes were heated in a water bath at 80 °C for 10 minutes (*Bo*H) or not heat treated (*Bo*NH). Cell material was collected and washed 4 times with phosphate-buffered saline and resuspended in sterile deionised water before being freeze-dried. The freeze-dried material was then resuspended in M2. Twenty µl of the freeze-dried material was added to a total volume of 200 µl and inoculated with *F. prausnitzii* at 2.5% (v/v).


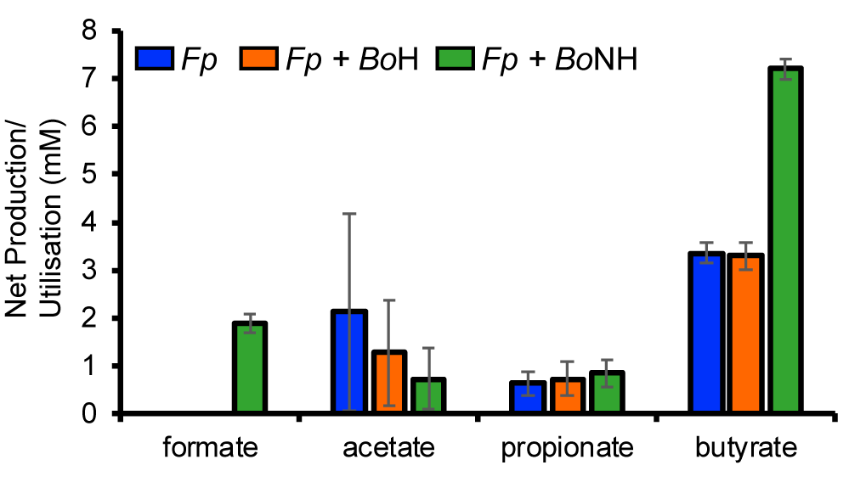

Supplement: FIG S3 [file mbio.03628-20-sf003.docx]
