# Supplementary material for: Human Gut Faecalibacterium prausnitzii Deploys a Highly Efficient Conserved System To Cross-Feed on β-Mannan-Derived Oligosaccharides
Source: mBio. 2021 Jun 1;12(3):e03628-20. doi: 10.1128/mBio.03628-20 (PMC8262883; doi:10.1128/mBio.03628-20)
Supplement: TABLE S2 [file mbio.03628-20-st002.docx]

**Table S2. qPCR primers used in this study with details on primer target, sequence, annealing temperature and references.**

| **Target** | **Name** | **Sequence (5’ - 3’)** | **annealing temp** | **Reference** |
| --- | --- | --- | --- | --- |
| All bacteria | UniF | GTGSTGCAYGGYYGTCGTCA | 60 °C | Chung et al. [1] |
|  | UniR | ACGTCRTCCMCNCCTTCCTC |  |  |
| *B. ovatus* | g-Bfra-R-Fmod | GCTCAACCKTAAAATTGCAGTTG | 63 °C | Chung et al. [1] |
|  | Bac708Rmod | GCAATCGGRGTTCTTCGTG |  |  |
| *R. intestinalis* | RrecF | GCGGTRCGGCAAGTCTGA | 63 °C | Chung et al. [1] |
|  | Rrec630mR | CCTCCGACACTCTAGTMCGAC |  |  |
| *F. prausnitzii* | FprauF | TGAGGAACCTGCCTCAAAGA | 63 °C | This study |
|  | FprauR | GACGCGAGGCCATCTCA |  |  |

1. Chung, W.S.F., et al., Prebiotic potential of pectin and pectic oligosaccharides to promote anti-inflammatory commensal bacteria in the human colon. FEMS Microbiol Ecol, 2017. **93**(11).
