# Supplementary material for: Human Gut Faecalibacterium prausnitzii Deploys a Highly Efficient Conserved System To Cross-Feed on β-Mannan-Derived Oligosaccharides
Source: mBio. 2021 Jun 1;12(3):e03628-20. doi: 10.1128/mBio.03628-20 (PMC8262883; doi:10.1128/mBio.03628-20)
Supplement: TABLE S1 [file mbio.03628-20-st001.docx]

**Table S1.** Primers used in this study.

| **Gene** | **Primer (5’ - 3’)** |
| --- | --- |
| FPR_17320 (*Fp*CE17) | **F:**TAAGAAGGAGATATACTATGAATACGTCTAATTTTGCTCGTTTGAAAG  **R:** AATGGTGGTGATGATGGTGCGCTGCCGTAATCAGAGCCATG |
| FPR_17300  (*Fp*CE2) | **F:** TTAAGAAGGAGATATACTATGGAGCAACTTACAACTGCGACG  **R:** AATGGTGGTGATGATGGTGCGCCAGGATCGTTCTCAGAAAGGC |
| FPR_17310  (*Fp*GH113) | **F:** TTAAGAAGGAGATATACTATGGAGCTGAAACATAACTTTTACGGG  **R:** AATGGTGGTGATGATGGTGCGCTTCTAAAACTCCTCCCTGATATGC |
| FPR_17200  (*Fp*GH36) | **F:** TTAAGAAGGAGATATACTATGATCGTCTGCACCGAAACTG  **R:** AATGGTGGTGATGATGGTGCGCTCACACTTTCTTCAAATACCATATC |
| FPR_17280 | **F:** TTAAGAAGGAGATATACTATGGGCTCCACCGGCAGCACC |
| (*Fp*MOBP) | **R:** AATGGTGGTGATGATGGTGCGCCTGCGGTGCGCAGGCATC |
